# Single whole genome sequencing analysis blazes the trail for precision medicine

**DOI:** 10.1080/15384047.2022.2033058

**Published:** 2022-02-06

**Authors:** Giulia C. Napoli, Cindy H. Chau, William D. Figg

**Affiliations:** Molecular Pharmacology Section, Genitourinary Malignancies Branch, Center for Cancer Research, National Cancer Institute, National Institutes of Health, Bethesda, MD, USA

**Keywords:** WGS, driver gene mutations, precision oncology, targeted therapies, biomarkers

## Abstract

As precision oncology evolves toward developing more targeted therapies, sequencing has moved to the forefront of treatment decision-making. Whole genome sequencing (WGS) has emerged as a technology capable of identifying candidates for rare and targeted treatments. Yet, because the tumor is constantly evolving during relapse and therapy resistance, the frequency with which WGS should be performed to identify potential new therapies for progressing patients remains unknown. A recent study in *Nature Medicine* by Van de Haar et al. observed a remarkably stable driver gene mutational profile among 250 biopsy pairs from 231 patients undergoing standard of care treatments during the biopsy interval. Their findings suggest that the actionable metastatic cancer genome is relatively stable over time and that a single WGS provides a complete view of the treatment opportunities available to most metastatic cancer patients.

The year 2021 marks the unveiling of the first truly complete human genome sequence, closing the gap on the final 8% of unexplored DNA missing from the initial publication of the sequence 20 years ago. This key milestone has ushered in the era of genomic medicine and initiated advances in various sequencing modalities from whole genome sequencing (WGS) to exome and targeted sequencing while cultivating major progress in many areas of medicine. WGS has become increasingly attractive in oncology as knowledge of the entire genetic code has advanced cancer diagnosis and treatment options, especially for rare, genomically driven cancers. Patients in genotype-matched trials and on molecular alteration-matched therapy have better treatment response compared to those on non-matched therapy.^1,2^ The approval of several tumor-agnostic and genotype-matched therapies has shifted the approach of precision oncology altogether, particularly with the implementation of master protocols (e.g., basket, umbrella, and platform trials) for evaluating biomarker-driven therapies, as in the NCI-MATCH trial.^3^

Unlike targeted next-generation sequencing (NGS) panels and exome sequencing, WGS provides a comprehensive profile of a tumor’s mutational landscape; yet, because many targeted therapies induce mutational changes,^4^ the frequency with which WGS should be performed remains unknown. A recent study by van de Haar and colleagues set out to answer the question of how significantly the actionable metastatic cancer genome changes during treatment.^5^ The whole genomes of 481 solid metastatic tumor biopsies from 231 adult patients and matched germline DNA were analyzed, allowing for 250 paired biopsy comparisons, with 6.4 months as the median time between biopsies (during which time patients were undergoing systemic therapy). At least one driver was detected in 97% of tumor samples, the number of drivers remained constant between paired biopsies, and 86% of drivers detected in the first biopsy were also detected in the second biopsy. Despite the relatively stable driver mutation profile, the cancer genome became more complex over time: the number of overall mutations per tumor, the number of genes affected by copy number alterations, and the genome wide number of structural variants significantly increased during the biopsy interval.

The authors next assessed the stability of genomic biomarkers that could determine standard of care (SOC) treatments, and 72% of samples had at least one such genomic biomarker. Full genomic biomarker concordance was observed in 99% of the biopsy pairs, suggesting limited evolution of the actionable metastatic tumor genome. In only three non-small cell lung cancer patients, did follow-up biopsies lead to the identification of a different SOC treatment. For 8.8% of cases, the second biopsy provided a new view of investigational treatment opportunities available to the patients. Meanwhile, 21% of cases treated with small-molecule inhibitors, and 22% of cases receiving hormonal therapy resulted in genomic alterations of the drug targets. Patients receiving small-molecule inhibitors or hormone therapy may benefit from repeat genomic evaluation due to the higher rate of genomic evolution stemming from these treatments.

The findings of this study suggest that a single WGS analysis should be performed early on in metastatic patients to identify all possible genome-matched treatments that more specific panels may not be able to inform, with the exception of patients receiving therapies that impose significant selective pressure and frequently induce on-target genomic evolution (e.g., hormonal therapy for prostate/breast cancer; small-molecule inhibitors for lung cancer). A recent prostate cancer case study revealed that truncal alterations identified in the primary tumor can drive advanced metastatic disease, even in the presence of additional oncogenes, supporting early detection and targeting of truncal alterations.^6^ Likewise, targeted sequencing data from the NCI-MATCH trial and TCGA suggest that a patient’s genetic landscape does not significantly evolve as they progress from primary to metastatic disease or under the pressure of cytotoxic chemotherapy.^3^ One limitation of the Van de Haar study is that it did not longitudinally compare the driver mutation profile between primary and metastatic sites and stands in contrast to another report, which found that the total number of late driver mutations in WGS samples from both primary and metastatic sites was similar to or greater than the number of early driver mutations.^7^ Furthermore, the coupling of WGS analysis with complementary deep sequencing of liquid biopsies will illuminate the mutational dynamics of disease progression. Current studies demonstrate that tissue-NGS and plasma-NGS are complementary and that tissue-NGS detects more clinically relevant alterations compared to plasma-NGS for certain tumor types.^8^ Tissue-NGS thus remains the preferred method until the clinical utility is validated and assay sensitivity is improved for plasma-NGS.^9^

Intratumoral heterogeneity calls into question the present findings as some drivers tend toward subclonality versus clonality, and some cancers are known to have higher subclonal mutational burdens than others.^10^ The effects of intra- and inter-tumoral heterogeneity aside cancer can evolve without genomic alterations. For instance, epigenetic reprogramming induces changes in chromatin and transcription, prompting major cellular changes. In a pancreatic ductal adenocarcinoma xenograft model in which the most common drivers of human PDAC (Kras gain of function and p53 loss of function) were employed to induce metastasis, 51% of clones failed to metastasize, implying that these driver mutations do not alone guarantee metastasis.^11^ Advancements in single cell genomics and transcriptomics coupled with CRISPR-Cas9 lineage tracing have the potential to shed light on driver mutation prevalence and changes, as well as transcriptomic dynamics. Likewise, the coupling of single cell RNA sequencing with spatial transcriptomics will clarify the tumor regions and subpopulations responsible for various aspects of tumor progression.^12^

In the two decades since the publication of the human genome, substantial progress has been made in understanding the complex genetic architecture of cancer and the application of genomics to diagnosing and treating the disease, as well as identifying potential drug targets. With advances in new DNA sequencing platforms and innovation in genome sequencing technologies, we can expect continued reductions in the cost of WGS and anticipate the identification of novel, clinically relevant variants arising from as-yet-unexplored areas of the genome. The next step forward is evaluation of the clinical utility of WGS in the selection and management of treatment options in larger cohorts of patients earlier in the disease state. Over a dozen new molecularly targeted drugs for precision oncology will emerge over the next decade, many of which will have tumor-agnostic implications. Incorporation of genomics knowledge from FDA-recognized human genetic variant databases (e.g., ClinGen, OncoKB) into evidence-based treatment paradigms can transform clinical care; yet, the question remains how best to implement WGS or comprehensive next-generation sequencing in routine clinical practice. Genomic medicine and the pharmacogenomics-based selection of the right drug for the right patient are here to stay as we continue to pave the way toward precision medicine.
